# Enhancing surgical decision-making in NEC with ResNet18: a deep learning approach to predict the need for surgery through x-ray image analysis

**DOI:** 10.3389/fped.2024.1405780

**Published:** 2024-06-04

**Authors:** Zhiqing Wu, Ran Zhuo, Xiaobo Liu, Bin Wu, Jian Wang

**Affiliations:** Department of Pediatric Surgery, Children’s Hospital of Soochow University, Suzhou, Jiangsu, China

**Keywords:** NEC, deep learning, pediatric surgery, diagnostic imaging, artificial intelligence in medicine

## Abstract

**Background:**

Necrotizing enterocolitis (NEC) is a severe neonatal intestinal disease, often occurring in preterm infants following the administration of hyperosmolar formula. It is one of the leading causes of neonatal mortality in the NICU, and currently, there are no clear standards for surgical intervention, which typically depends on the joint discretion of surgeons and neonatologists. In recent years, deep learning has been extensively applied in areas such as image segmentation, fracture and pneumonia classification, drug development, and pathological diagnosis.

**Objective:**

Investigating deep learning applications using bedside x-rays to help optimizing surgical decision-making in neonatal NEC.

**Methods:**

Through a retrospective analysis of anteroposterior bedside chest and abdominal x-rays from 263 infants diagnosed with NEC between January 2015 and April 2023, including a surgery group (94 cases) and a non-surgery group (169 cases), the infants were divided into a training set and a validation set in a 7:3 ratio. Models were built based on Resnet18, Densenet121, and SimpleViT to predict whether NEC patients required surgical intervention. Finally, the model's performance was tested using an additional 40 cases, including both surgical and non-surgical NEC cases, as a test group. To enhance the interpretability of the models, the study employed 2D-Grad-CAM technology to describe the models’ focus on significant areas within the x-ray images.

**Results:**

Resnet18 demonstrated outstanding performance in binary diagnostic capability, achieving an accuracy of 0.919 with its precise lesion imaging and interpretability particularly highlighted. Its precision, specificity, sensitivity, and F1 score were significantly high, proving its advantages in optimizing surgical decision-making for neonatal NEC.

**Conclusion:**

The Resnet18 deep learning model, constructed using bedside chest and abdominal imaging, effectively assists clinical physicians in determining whether infants with NEC require surgical intervention.

## Introduction

NEC is the leading cause of severe inflammatory disease in newborns post-birth (1), especially among preterm infants, and is a principal contributor to neonatal mortality in the NICU (2). Newborns suffering from NEC, particularly after complications like intestinal perforation and peritonitis (3), often experience worsening conditions necessitating transfer to specialized pediatric hospitals equipped for surgery. In such instances, mortality rates can soar to 30%, and even 50% due to widespread intestinal necrosis. Additionally, up to 9% of children at the age of one who have had NEC require parenteral nutrition (3), and many post-surgery infants exhibit intestinal flora dysbiosis (4). The rapid progression of NEC sometimes calls for multiple short-term assessments to decide on the urgency of surgical intervention (5). Surgical management of NEC aims to mitigate further infection (6) and sepsis by repairing perforations and resecting non-viable bowel sections (6, 7), which is crucial for preventing short bowel syndrome (8) and the prolonged use of parenteral nutrition. Currently, there are no definitive standards for surgical indications, which often rely on the joint discretion of surgeons and neonatologists (9). The presence of free air under the diaphragm on an upright abdominal x-ray and progressive abdominal distension are the gold standards for surgical intervention in acute NEC. Clinically, the ideal surgical window during the acute phase of NEC—when the intestinal wall is fully necrotic but not yet perforated—is highly sought after. Furthermore, severe intestinal strictures post-conservative treatment still warrant surgical indications.

In recent years, deep learning has seen extensive application in diagnosing traumatic bone fractures (10), pulmonary nodules (11), COVID-19 (12), and in classifying pneumonia as well as in image segmentation (13, 14). Deep learning neural networks, by mimicking the human brain, automatically learn and recognize patterns in images, offering an edge in processing complex image data (15). Currently, the use of supine x-ray films in NEC lacks a standardized approach (16). In our research, we have trained deep learning models based on bedside chest-abdominal x-rays using Densenet121, Resnet18, and SimpleViT. Our study underscores ResNet18's potential to refine NEC surgical decision-making. These models are commonly employed in pneumonia classification (11, 17) and the identification of gastrointestinal pathologies (18).

## Materials and methods

### Materials

From January 2015 to April 2023, bedside chest and abdominal films of 263 neonates aged 0 to 39 days were collected from Children's Hospital of Soochow University for this study. We divided these original images into a 7:3 ratio for training and validation. These infants were treated for NEC either surgically or non-surgically. In addition, we have collected 40 cases of non-surgical and surgical NEC from May 2023 to February 2024 as an independent validation set to assess the model's performance on unfamiliar datasets. Additionally, from May 2023 to February 2024, we collected a total of 40 cases, including 21 non-surgical and 19 surgical NEC cases, as an independent validation set to evaluate the model's performance on unfamiliar datasets. The diagnosis of the non-surgical group was based on the modified Bell staging criteria, and the initial bedside chest and abdominal films in the anteroposterior position, decided for conservative treatment, were used. For the surgical group, the last bedside chest and abdominal films in the anteroposterior position before surgery, which were confirmed as NEC during the surgery, were used. The bedside chest and abdominal films were acquired using the DRX-Revolution Mobile x-Ray System produced by Carestream Health, USA. All images were taken in the supine position. According to the needs of the disease, some patients also underwent anteroposterior and lateral CAXR, but our study did not involve lateral views. As part of a retrospective study, we initially collected a large volume of clinical diagnostic NEC x-ray images from cases that did not undergo surgical treatment for model training. These images were subsequently annotated by experienced pediatric radiologists to select those with relatively distinct radiographic features. For the non-surgical group, images taken prior to antibiotic treatment were used; for the surgical group, images from the last bedside review before surgery were utilized. Surgical indications include cases where the child's upright abdominal x-ray shows signs of pneumoperitoneum, abdominal paracentesis fluid indicates the presence of feces or a large amount of purulent or bloody fluid, and situations where, despite receiving the best medical treatment, the condition continues to worsen or remains unstable. Surgical interventions for these cases involved simple laparotomy, and if necrosis was discovered, resection of the necrotic bowel segments followed by the establishment of an intestinal stoma or anastomosis. Cases diagnosed with NEC preoperatively were included; however, images from cases not confirmed as NEC postoperatively were excluded to enhance the model's diagnostic specificity. The inclusion criteria included (1)NEC patients are diagnosed and treated according to the modified Bell staging.(2) sufficient radiographic image technical quality, (3) imaging field of view (FOV) covering the entire abdomen.(4)During the surgery and in the postoperative pathology, it was confirmed that the children who underwent surgical treatment had NEC. Exclusion criteria include: children with congenital intestinal malformations (congenital megacolon, intestinal atresia, malrotation of the intestines), meconium ileus, spontaneous intestinal perforation, and cases with a large amount of incomplete data. The diagnosis of NEC was confirmed by three senior pediatric radiologists and neonatologists with over five years of experience.

The study was conducted with the consent of the parents of the children and was approved by the Institutional Ethics Committee of Children's Hospital of Soochow University.

## Methods

Data augmentation, commonly used in the medical field to increase the size of the dataset, generates additional labeled images without changing the semantics of the image. In this paper, we used various data augmentation methods, such as random cropping, rotation, and horizontal flipping. In implementation, the CPU generates augmented images while the previous batch of images is being trained on the GPU. Thus, these data augmentation techniques do not affect the time complexity. We also used oversampling to deal with imbalanced data. Deep learning methods automatically extract features from raw data and classify images. The main advantage of this approach is that both feature extraction and classification occur within the same network. Convolutional Neural Network (CNN) models, the most advanced form of DL technology, consist of many stacked convolutional layers that automatically extract features from image data. They have been used in many radiological tasks and can achieve high performance in image-based disease classification (19). The CNN architecture is built with layers including an input layer (producing output from the image as input), convolutional layers (convoluting the input image with filters to produce feature maps), Rectified Linear Unit (ReLU) activation layers (activating neurons above a threshold), pooling layers (reducing the image size while retaining high-level features), and fully connected (FC) layers (producing results) (8). The accuracy of CNNs depends on the design of the layers and the training data. CNNs typically require large labeled medical datasets for training, which are difficult to create due to time and labor costs. Recent studies have shown that transfer learning can address issues with small datasets.

In transfer learning (TL), a Convolutional Neural Network (CNN) is first trained to learn features in a broad domain (e.g., ImageNet), then the trained features and network parameters are transferred to a more specific domain. In CNN models, low-level features like edges, curves, and corners are learned in initial layers, while specific high-level features are learned in the final layer (20). Among different TL models, we chose ResNet for its widespread recognition in medical image classification. We used ResNet-18 due to its relatively shallow structure, allowing faster training of images without sacrificing performance. It consists of a 7 × 7 convolutional layer, 2 pooling layers, 5 residual modules, and a fully connected (FC) layer. Each residual module contains two 3 × 3 convolutional layers followed by a batch normalization layer and a ReLU activation function. Inputs can be added directly before the final ReLU activation function by skipping these two convolutional layers. In a recent study (21), the authors evaluated the performance of several neural networks with a Softmax output layer with a ReLU activation layer and verified that Softmax with ReLU activation performs better in classification tasks. Therefore, Softmax was preferred as the output layer in this study to obtain probability predictions.

Due to its use of bottleneck residual blocks, batch normalization (adjusting the input layer), and identity connections (to prevent gradient vanishing in the network), it has high classification accuracy. During the transfer learning and fine-tuning process, we gradually unfroze the top 10 layers of the model and changed the output of the fully connected (FC) layer to binary classification. We tested various optimizers and found the “Adam” optimizer (22) to perform best among all studied optimizers, hence it was applied in our model. To our knowledge, no previous studies have fevaluated the effectiveness of these deep learning models that can directly recognize bedside chest-abdominal x-ray images without the need for delineating regions of interest (ROI) in assessing the need for surgical intervention.

All methods were carried out in accordance with relevant guidelines and regulations. Informed consent was obtained from all legal guardians of the patients. Personal identifiers were removed from all patient data to protect privacy and confidentiality.

### Comparison of deep learning models

In this study, we explored which deep learning model had superior performance in determining the need of NEC surgery by comparing three commonly used deep-learning-based classification methods: ResNet-18 (a 18-layer residual network), DenseNet, and the latest Transformer model architecture, which integrates multi-head attention mechanisms (MHA) in two schemes to enhance model performance. Initially, we conducted a comparative analysis of our ResNet18 model against the deeper ResNet50 and ResNet101 models to evaluate their performance. Surprisingly, we found that the latter two models underperformed, whereas the ResNet18 model demonstrated superior predictive capabilities for our research objectives. Additionally, we employed the 2D-Grad-CAM (23) module for interpretability analysis of the three deep learning models. This module allows for a visual identification of the alignment between intestinal lesions and model prediction focus. We also utilized Decision Curve Analysis (DCA) to evaluate the clinical benefits of different prediction models.

### Statistical analysis

We use the following indicators to evaluate the performance of the model and select the best model: accuracy, sensitivity, specificity, F1 score, DCA curve images, where the F1 score is the weighted average of precision and recall. This study used the following tools: Python 3.7.16 (https://www.python.org/downloads/release/python-3716/) and PyTorch third-party libraries (Version: 1.13.1) on Windows 11 operating system {[MSC v.1916 64 bit (AMD64)]}.

## Results

**Figure 1:**
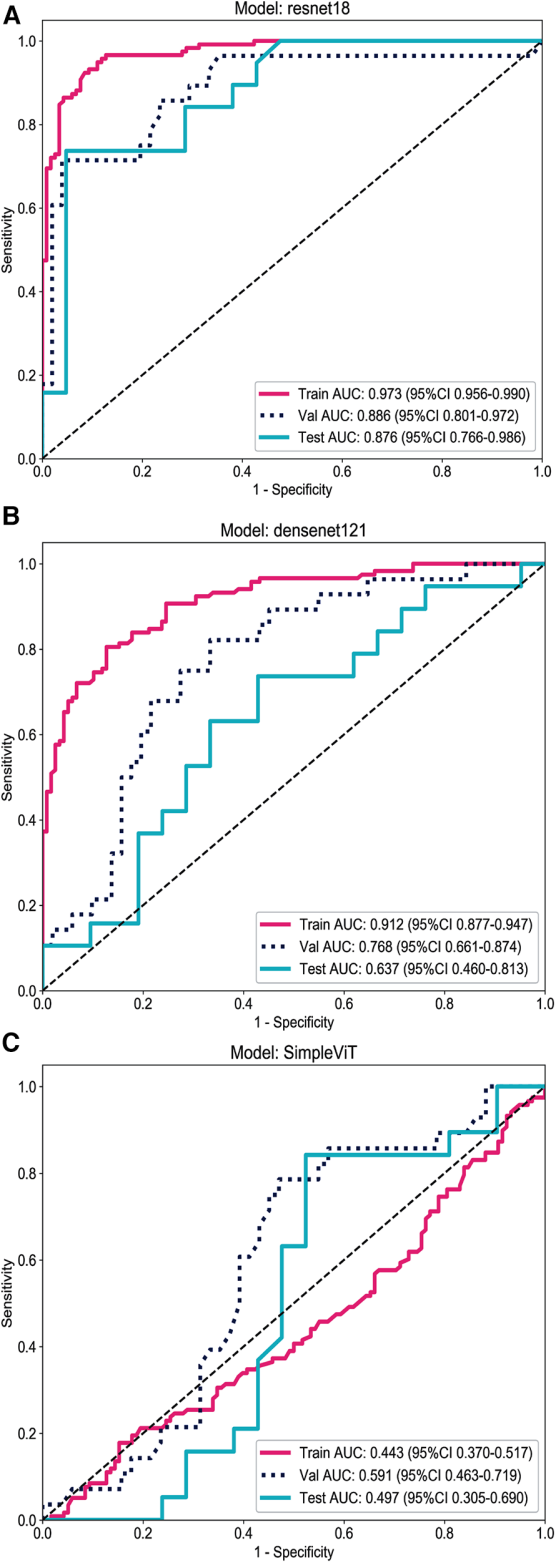
Comparison of the AUC curves for the three models across the training, validation, and testing datasets. (**A**) Resnet18. (**B**) Densenet121. (**C**) SimpleViT.

In terms of AUC curve performance (Figure 1), the Resnet18 model demonstrates superior classification capability compared to the Densenet121 and SimpleViT models, and it maintains high accuracy on unseen datasets.

As shown in Figure 1A, the Resnet18 model has an AUC of 0.973 on the training set, indicating a high level of classification efficacy. The AUC on the test set is 0.876; although it decreases compared to the training set, it still reflects the model's robust classification capability.

**Figure 2:**
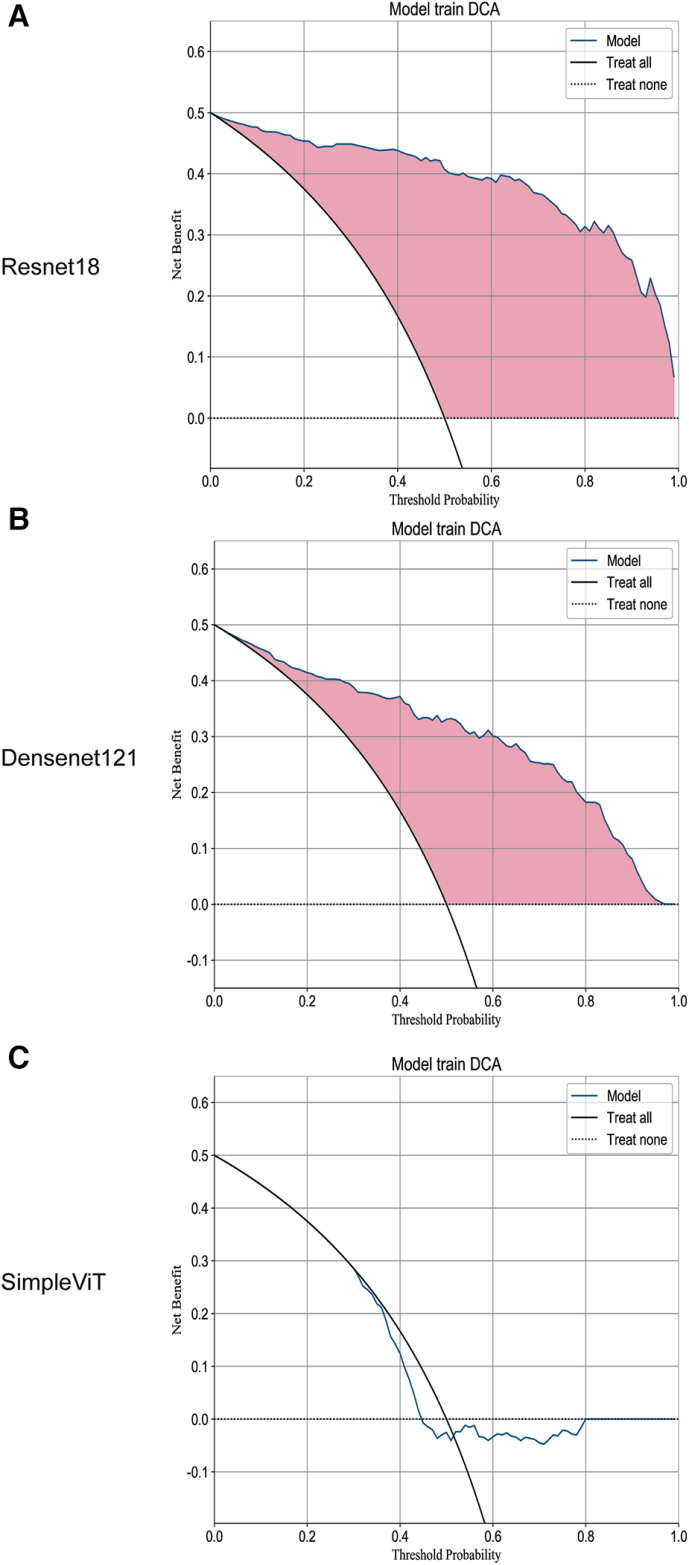
The DCA curves of the three models on the training set. (**A**) Resnet18. (**B**) Densenet121. (**C**) SimpleViT.

**Figure 3:**
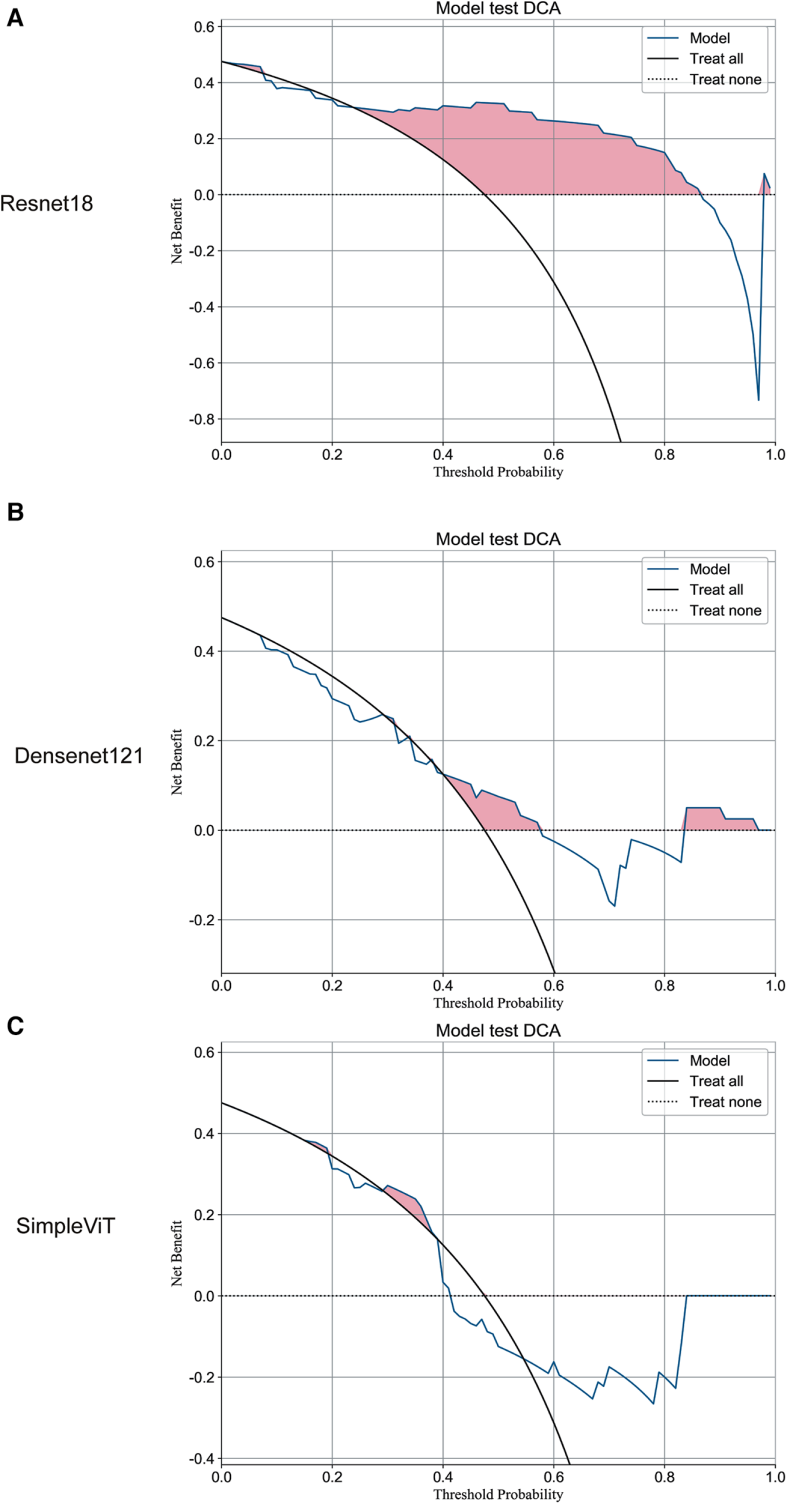
The DCA curves of the three models on the test set. (**A**) Resnet18. (**B**) Densenet121. (**C**) SimpleViT.

**Figure 4:**
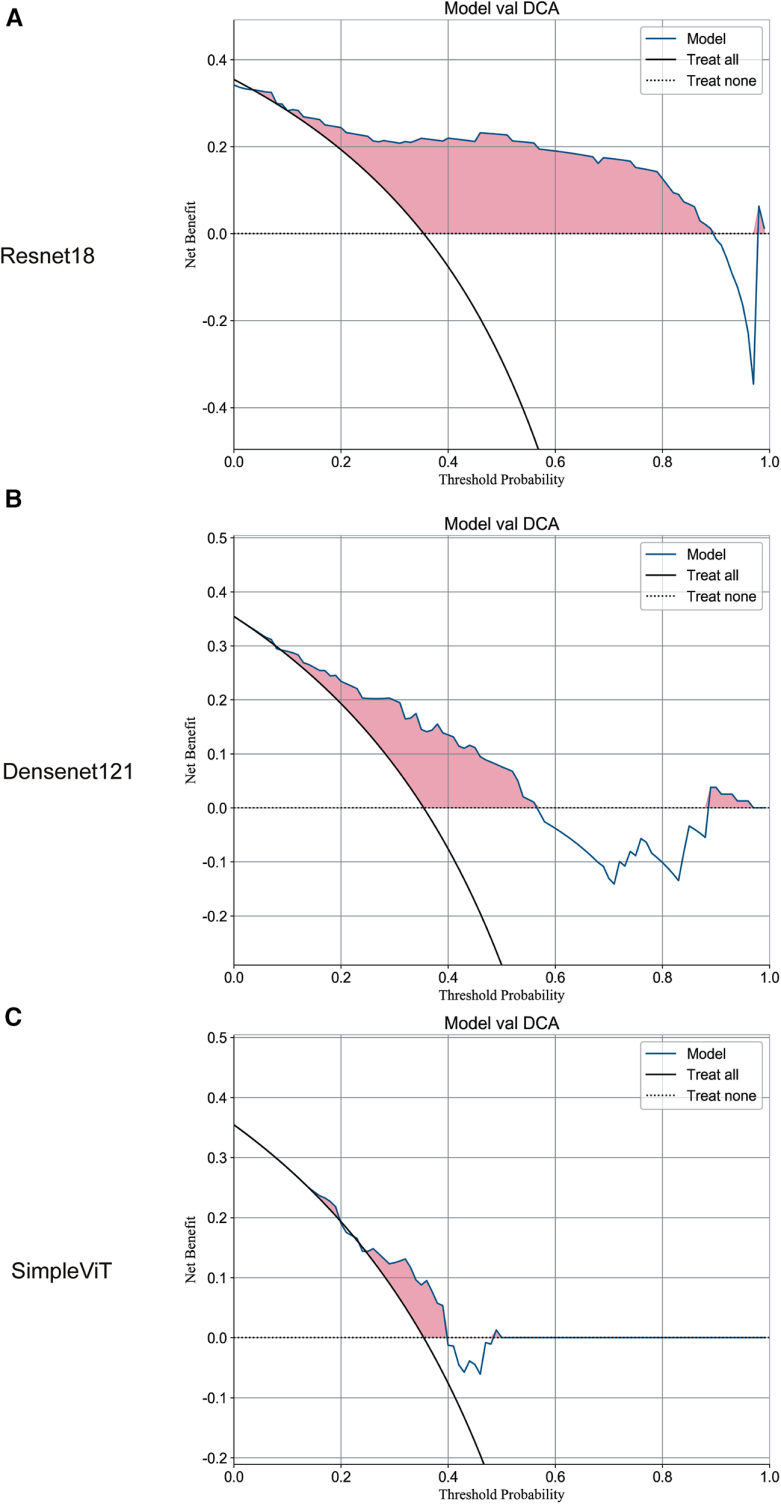
The DCA curves of the three models on the validation set. (**A**) Resnet18. (**B**) Densenet121. (**C**) SimpleViT.

Additionally, the Resnet18 model demonstrated good performance in predictive decision applications, outperforming both the Densenet121 and SimpleViT models across training (Figure 2), testing (Figure 3), and validation sets (Figure 4).

As shown in Figure 2A, on the training set, the Decision Curve Analysis (DCA) performance of the Resnet18 model consistently outperforms the “treat none” strategy. Moreover, at most threshold values, the model's curve exceeds the “treat all” approach, suggesting that this model could assist clinicians in making accurate decisions about whether to proceed with surgical interventions in children diagnosed with NEC.

**Figure 5:**
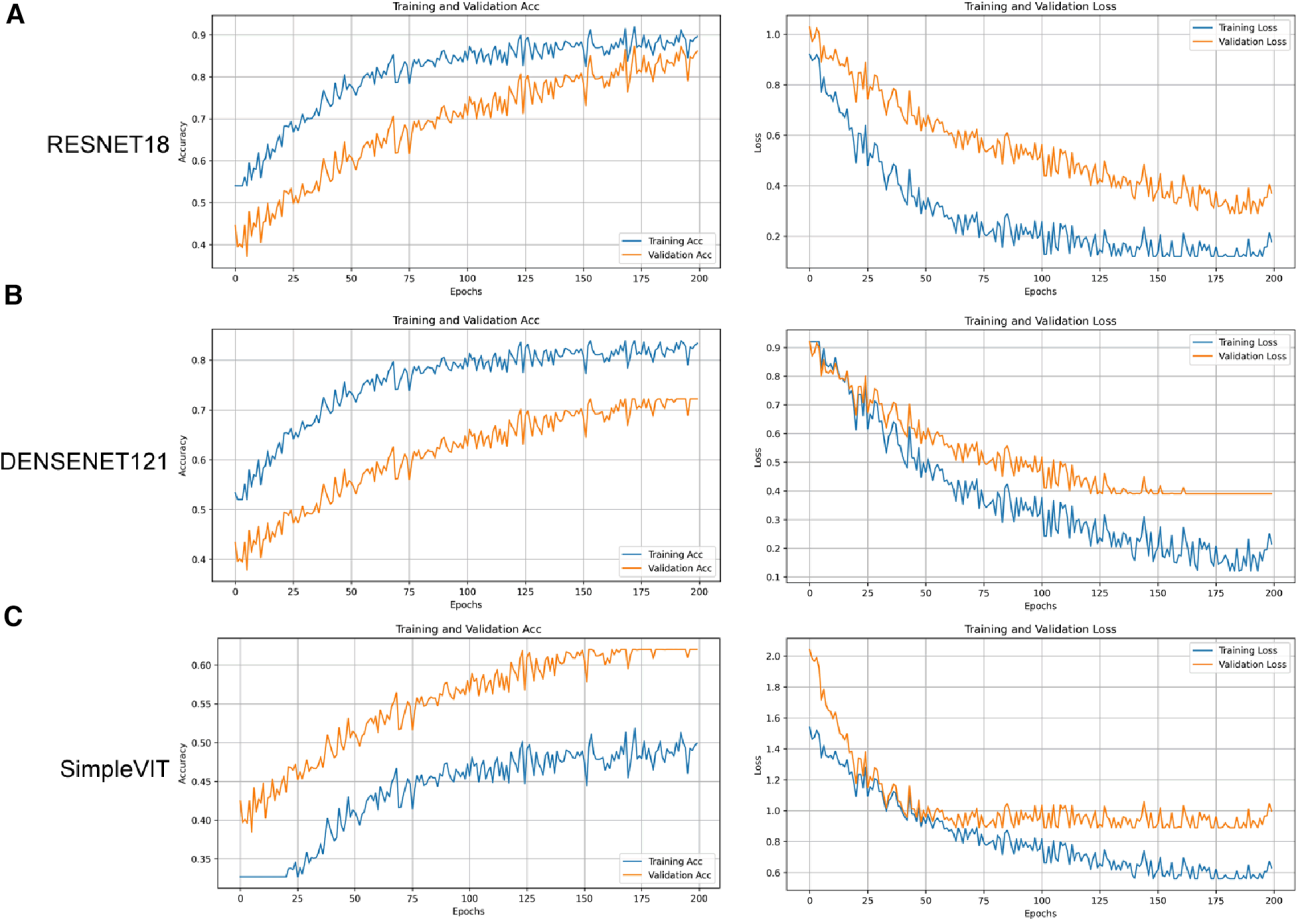
The accuracy and loss curves for the three models on the training and validation sets. (**A**) Resnet18. (**B**) Densenet121. (**C**) SimpleViT.

As illustrated in Figure 5A, compared to Densenet121 (Figure 5B) and SimpleViT (Figure 5C), the Resnet18 model demonstrates good performance on the training set and exhibits a positive learning and generalization trend on the validation set, although the latter's rise is not as steady as on the training set. With the number of training iterations increasing, the loss curves for both the training and validation sets demonstrate a declining trend, indicating that the model is progressively enhancing its predictive accuracy while minimizing loss as much as possible.

Overall, the Resnet18 model exhibits excellent performance on the training set and maintains good performance on the test set, despite an expected decrease in effectiveness as the model transitions from familiar training data to previously unseen data. However, it is noteworthy that the AUC value for the test set indicates the model's potential for providing robust diagnostic or predictive capabilities in practical applications.

Table 1 displays a comparative analysis of various performance metrics for three models - Densenet121, Resnet18, and SimpleViT—across training, validation, and test datasets. These metrics include accuracy (Acc), area under the curve (AUC), 95% confidence interval (95% CI), sensitivity, specificity, positive and negative predictive values (PPV and NPV), precision, recall, F1 score, and decision threshold. The Resnet18 model exhibited robust performance with high accuracy and AUC, indicating strong generalizability.

**Table 1:** Comparison of the performance of five deep learning models.

Model Name	Acc	AUC	95% Cl	SENS	SPE	PPV	NPV	Precision	Recall	F1	Threshold	Cohort
Densenet121	0.839	0.912	0.877–0.948	0.805	0.873	0.864	0.817	0.864	0.805	0.833	0.519	TRAIN
	0.722	0.768	0.661–0.874	0.821	0.667	0.575	0.872	0.575	0.821	0.676	0.313	Val
	0.65	0.637	0.460–0.813	0.737	0.571	0.609	0.706	0.609	0.737	0.667	0.313	TEST
Resnet18	0.919	0.973	0.956–0.990	0.924	0.915	0.916	0.923	0.916	0.924	0.92	0.494	TRAIN
	0.873	0.886	0.801–0.972	0.714	0.961	0.909	0.86	0.909	0.714	0.8	0.513	Val
	0.85	0.876	0.766–0.986	0.737	0.952	0.933	0.8	0.933	0.737	0.824	0.513	TEST
SimpleViT	0.513	0.443	0.370–0.517	0.178	0.855	0.538	0.508	0.538	0.178	0.268	0.56	TRAIN
	0.62	0.591	0.463–0.719	0.786	0.529	0.478	0.818	0.478	0.786	0.595	0.36	Val
	0.65	0.497	0.305–0.691	0.842	0.5	0.593	0.769	0.593	0.842	0.696	0.36	TEST
Resnet50	0.924	0.907	0.861–0.954	0.907	0.941	0.939	0.91	0.939	0.907	0.922	0.501	TRAIN
	0.65	0.622	0.435–0.808	0.842	0.476	0.593	0.769	0.593	0.842	0.696	0.379	VAL
	0.722	0.661	0.533–0.789	0.321	0.941	0.75	0.716	0.75	0.321	0.45	0.481	TEST
Resnet101	0.919	0.973	0.957–0.990	0.915	0.924	0.923	0.916	0.923	0.915	0.919	0.498	TRAIN
	0.675	0.638	0.456–0.820	0.842	0.524	0.615	0.786	0.615	0.842	0.711	0.142	VAL
	0.595	0.587	0.460–0.714	0.857	0.451	0.462	0.852	0.462	0.857	0.6	0.128	TEST

Furthermore, through 2D-Grad-CAM analysis applied to the final convolutional layer of the model (Supplementary Material Figures S1–S3), heatmaps highlighting focal areas were generated. This study provides visual insigts into the model's capability to detect intestinal lesions. Notably, the Resnet18 model exhibited significant alignment and activation in the intestinal regions, emphasizing its interpretative accuracy in medical imaging diagnosis.

## Discussion

Previous studies, such as the one by Gao et al. (16), developed a complex multimodal model based on radiomic features and the SENet network model to predict surgical necessity in acute NEC cases. However, this did not include NEC cases requiring surgical intervention after treatment. In other aspects, many studies have described several potential biomarkers, mainly isolated from serum, stool, and urine samples, to differentiate between non-surgical and surgical NEC. Cakir et al. (24) described the predictive value of endothelial cell-specific molecules and interleukin-33, which gradually increase in patients undergoing surgery. However, obtaining these indicators is time-consuming and expensive, often necessitating surgical intervention in patients before detection, showing a clear lag compared to bedside chest and abdominal x-rays. In ultrasonography diagnosis, Chen QinYao et al. (9) described the role of focal or strong echo effusion in surgical prediction. However, ultrasonography often depends on the level and subjective judgment of the ultrasonographer, lacking objectivity compared to x-ray examination and is greatly interfered by increased intestinal gas. Currently, bedside ultrasound or upright abdominal films are more commonly used in clinical practice for intestinal condition analysis, with few using abdominal CT scans for evaluation. However, in our study, we decided to use bedside chest and abdominal films to construct models. Performing upright abdominal films usually requires a DR photography room and is inconvenient for bedside operation. Bedside chest and abdominal films are convenient, low-cost, highly repeatable, and easy to master, providing advantages for diseases like NEC that may require multiple assessments within a day. Moreover, bedside chest and abdominal films, compared to ultrasound, provide more objective and comprehensive results. Also, compared to abdominal CT scans, bedside chest and abdominal films involve lower radiation doses, making them more feasible for generally unstable infants and having lower side effects. For the non-surgical group, the most significant imaging features of NEC can be obtained, and for the surgical group, bedside film images closest to the clinical physician's judgment standard are obtained. We constructed an auxiliary means for determining the treatment strategy for neonatal NEC using three deep learning models (Densenet121, Resnet18, SimpleVit) and analyzed and compared their efficacy. Table 1 indicates that the Resnet18 model outperforms the other two models in terms of DCA and AUC curves, as well as 2D-Grad-CAM. It exhibits high values in sensitivity, specificity, PPV, and NPV across training, validation, and test datasets. Furthermore, by fine-tuning hyperparameters, we achieved superior performance compared to the conventional Resnet18 model. Notably, the model demonstrates excellent generalization capability on the validation set. Hence, we believe this model could aid clinicians in deciding whether surgical intervention is necessary for NEC patients, thus preventing the risk of missing the optimal surgical window due to continued conservative treatment.

### Limitations

It is a single-center study with a relatively small sample size. Moreover, assessing whether children require NEC surgery relies not only on x-ray examinations but also necessitates an analysis of the infant's basic vital signs and tolerance for surgical intervention and general anesthesia. In the clinical decision-making process, other clinical indicators such as complete blood count, coagulation tests, blood gas analysis, and electrolyte levels are indispensable. Looking ahead, we plan to collaborate with multiple centers to collect more bedside chest and abdominal x-ray images of infants with NEC, aiming to enlarge the scale of the training, validation, and test sets. We will also explore adjustments in fine-tuning methods to enhance accuracy, such as modifying the model's loss function, learning rate, employing different optimizers, and implementing early stopping mechanisms.

## Conclusion

The Resnet18 deep learning model, constructed using bedside chest and abdominal imaging, effectively assists clinical physicians in determining whether infants with NEC require surgical intervention.

## Data Availability

The original contributions presented in the study are included in the article/**Supplementary Material**, further inquiries can be directed to the corresponding authors.

## Supplementary material

Supplementary Figure S1

The regions of interest activated in the training set by the 2D-Grad-CAM module during the prediction of supine abdominal plain films by three different models.

Supplementary Figure S2 AND S3

The attention areas in the prediction of the Resnet18 model for supine abdominal radiographs of surgical versus non-surgical NEC within the test group activated by the 2D-Grad-CAM module.

Supplementary Figure S4

The calibration curves for each model of RESNET18, DENSENET121 and SimpleVIT.

Supplementary Figure S5

Comparison of the performance of the ResNet50 and ResNet101 models.
