# Supplementary material for: Enhancing surgical decision-making in NEC with ResNet18: a deep learning approach to predict the need for surgery through x-ray image analysis
Source: Front Pediatr. 2024 Jun 4;12:1405780. doi: 10.3389/fped.2024.1405780 (PMC11183801; doi:10.3389/fped.2024.1405780)
Supplement: Supplementary file 1 [file Table1.pdf]

**Supplementary Table 1.** The Baseline Information of Patients.

| Baseline Information of Patients | | | | | | | |
|---|---|---|---|---|---|---|---|
| | Hospital Stay (days) | Gestation Period (weeks) | | Birth Weight (grams) | | Age when NEC (weeks) | Weight when NEC (grams) |
| Groups and cases | Mean | Mean | Std Dev | Mean | Std Dev | Mean | Mean |
| Surgical (113) | 39.7 | 31.02 | 4.58 | 1653.42 | 2110.22 | 33.53 | 1813.95 |
| Non-Surgical（190） | 26.97 | 34.08 | 3.7 | 819.05 | 825.01 | 35.62 | 2168.61 |
