# Supplementary figures and images for: Enhancing surgical decision-making in NEC with ResNet18: a deep learning approach to predict the need for surgery through x-ray image analysis
Source: Front Pediatr. 2024 Jun 4;12:1405780. doi: 10.3389/fped.2024.1405780 (PMC11183801; doi:10.3389/fped.2024.1405780)

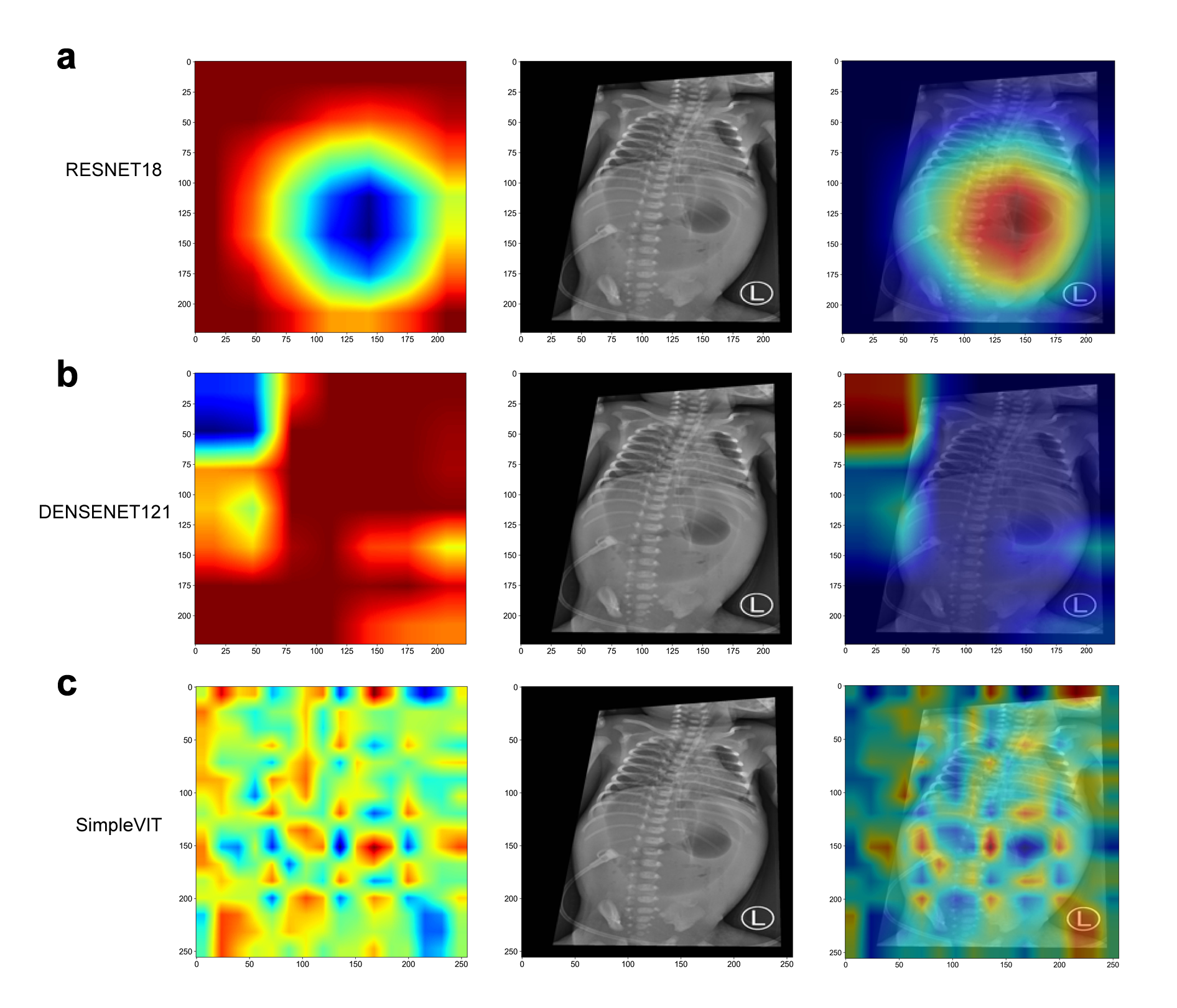

Supplement: Supplementary file 2 [file Image1.tiff]

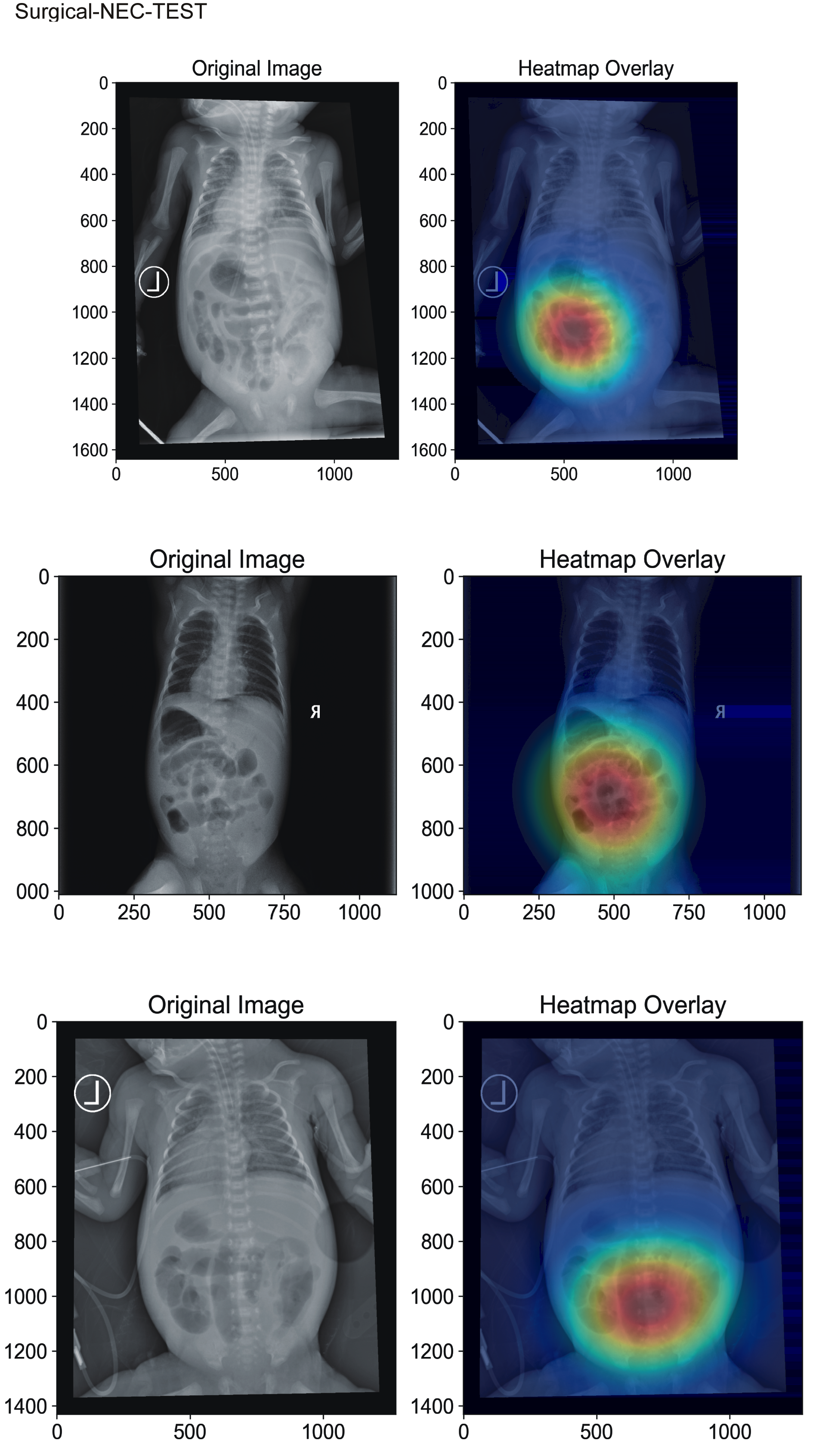

Supplement: Supplementary file 3 [file Image2.tif]

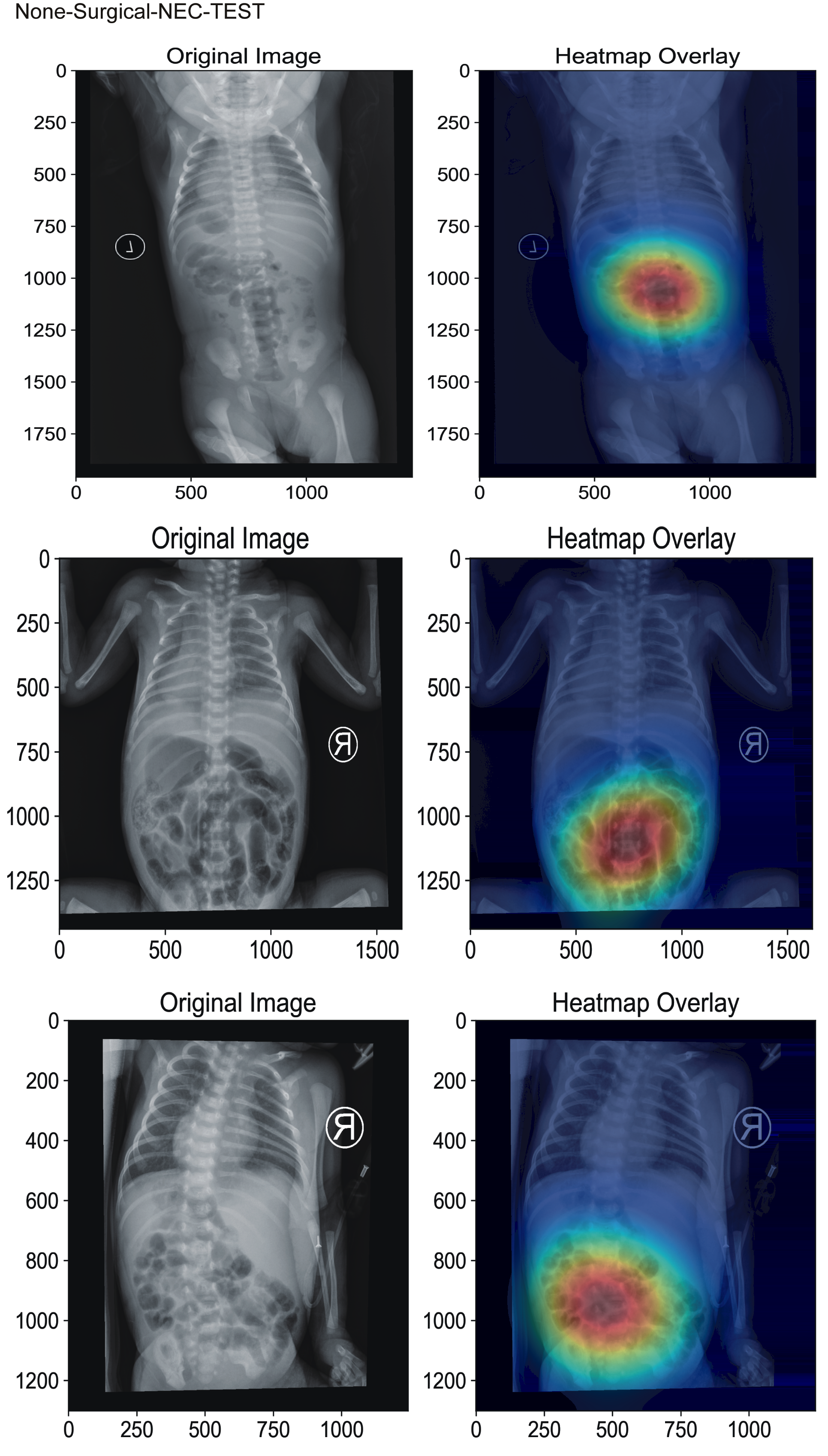

Supplement: Supplementary file 4 [file Image3.tif]

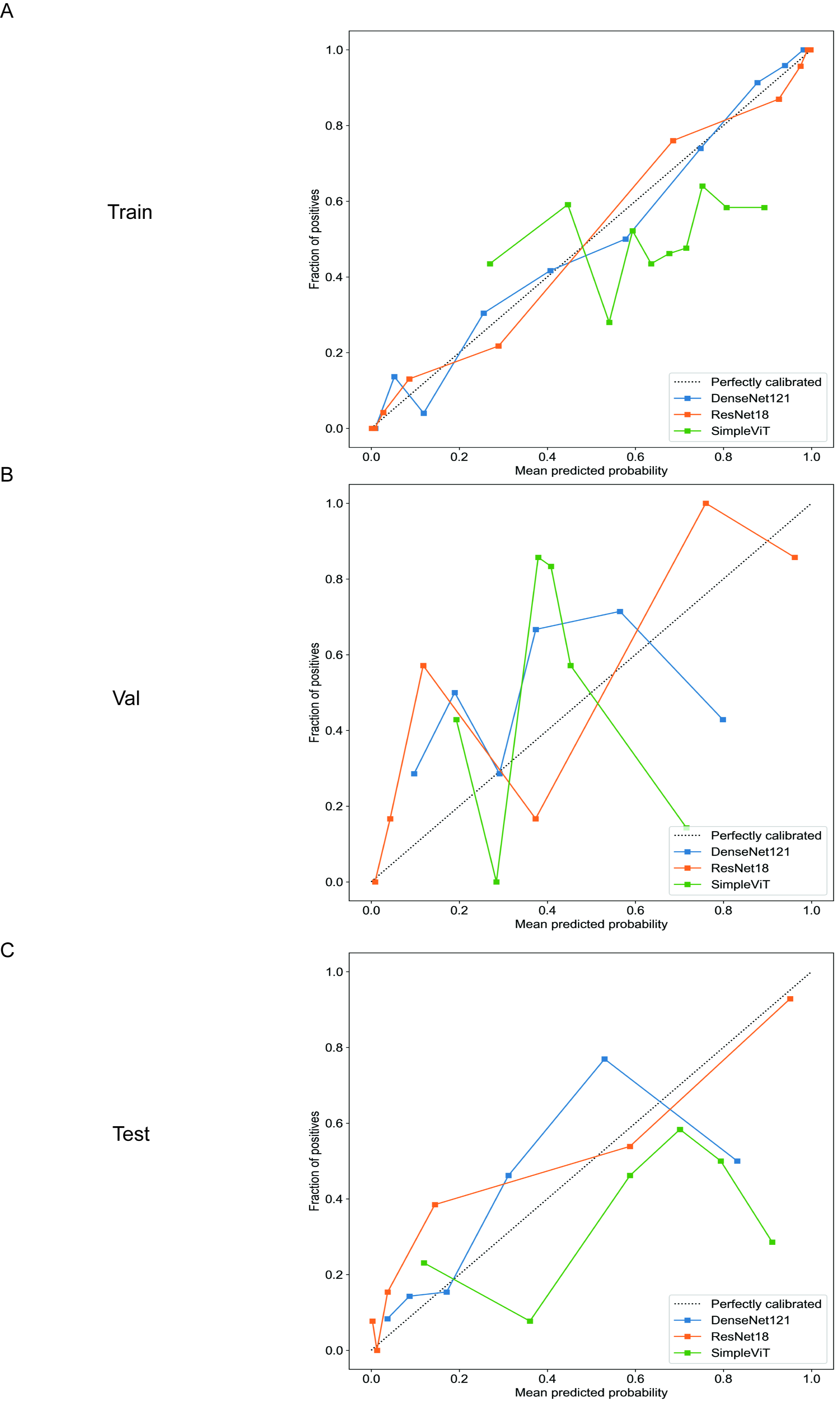

Supplement: Supplementary file 5 [file Image4.tif]
